# Evolution of negative immune regulators

**DOI:** 10.1371/journal.ppat.1007913

**Published:** 2019-08-01

**Authors:** Steven A. Frank, Paul Schmid-Hempel

**Affiliations:** 1 Department of Ecology and Evolutionary Biology, University of California, Irvine, California, United States of America; 2 Institute of Integrative Biology, ETH Zürich, Zürich, Switzerland; University of Pittsburgh, UNITED STATES

Hosts must recognize pathogen invasion and respond rapidly. The need for speed requires a low threshold for triggering a response, causing occasional false alarms. Host immune systems therefore require strong negative regulators to shut down unnecessary responses. The evolutionary consequences of rapid trigger dynamics balanced by negative regulators have received little attention. Here, we emphasize four aspects that influence the evolutionary genetics of immunity: diverse gene families of rapidly acting triggers opposed by slower-acting negative regulators, pathogen attack against negative regulators, diversifying selection of negative regulators by pathogen pressure, and heritability of immune-related disease from imbalance between triggers and negative regulators.

## Background on triggers of immunity

A broad sensor array of receptors, located on the cell surface or in the cytosol, recognize pathogen-associated molecular patterns and trigger innate immunity. Common triggers include the peptidoglycan recognition proteins, gram-negative binding proteins, Toll-like receptors (TLRs), nucleotide-binding domain leucin-rich repeats (NLRs), and the cyclic GMP-AMP synthase (cGAS) system that detects double-stranded DNA (dsDNA) [1, 2]. In the NLR family of immune triggers, some plant species have more than 1,000 genes [3], the coral *Acropora digitifera* has around 500 genes, sea urchins have approximately 200, and zebrafish have about 385 [4]. Sponges, perhaps the most ancient animals, have a diverse family of NLR receptors that can recognize a wide variety of microbes [5].

These receptors trigger immune cascades that are often evolutionarily ancient within groups or across broad domains of life. Key components of such cascades include nuclear factor-κB (NF-κB) transcription factors, interferons (IFNs), interleukins, and Toll and immune deficiency (IMD) pathways [6–9]. The primary dsDNA sensor cGAS triggers stimulator of IFN genes (STING), which activates a variety of fundamental immune cascades [10].

## Prediction 1: Fast, error-prone triggers of innate immunity are balanced by slower-acting negative regulators that provide a more accurate assessment of invasion

The need for speed in immune response [11] demands a low threshold for triggering the immune cascade, which inevitably leads to many errors. The evolution of the trigger threshold will be influenced by the risk of attack and the costs and benefits defense [12]. The faster and more error-prone the initial triggers, the greater the need for negative regulators that can correct errors.

All organisms have an impressive array of negative regulators. Post-transcriptional modification of innate sensors and downstream molecules suppress the effect of the pattern recognition receptor triggers [13, 14]. Tripartite motif containing 25 (TRIM25) negatively regulates the innate antiviral responses [15]. Various negative regulators suppress the Toll cascade and cytokine-signaling in mammals [16], including type I IFN [17]. The cascade triggered by the dsDNA sensor cGAS has several negative regulators, perhaps because dsDNA is nonspecific and prone to false signaling [10]. Numerous noncoding micro-RNAs (miRNAs) regulate innate immunity, including the Toll cascade in defense against gram-negative bacteria [18], modification of type I IFN regulation [17, 19], immune cell differentiation [20], and repression of the NLR triggers and their immune cascade [21, 22].

Different organisms will tune the tradeoff between faster response and lower error rates in different ways. A focus on the tuning of that tradeoff may lead to interesting comparative insights about the design of immune regulation.

## Prediction 2: Negative regulators of immunity provide a point of attack for invaders

Attackers use diverse mechanisms to block the expression of host innate immunity [23, 24, 25–27]. We predict that attackers may often target stimulation of the host’s natural negative immune regulators. In fact, *Leishmania* exploits a negative TLR regulator to suppress production of key proinflammatory cytokines [28], viruses manipulate post-translational modifications that normally prevent aberrant responses [29], and many microbes interfere with (negative) receptor crosstalk that normally adjusts immune responses [30].

Invaders of insects alter the expression of an impressive variety of miRNAs, including negative regulators. Some pathogens express their own miRNAs to alter host immunity. The function of most miRNAs remains unknown, providing a rich topic for further study [31].

Tumors also evolve to down-regulate immunity against cancer cells and often manipulate the normal repressors of immunity. For example, Vinay and colleagues [32] note that “tumors can evade immune surveillance by … production of several immune suppressive cytokines … In addition to immune suppressive cytokines, other factors … inhibit the differentiation of progenitors … affecting efficient uptake and antigen presentation.” The multiple ways in which tumors manipulate negative regulators of immunity suggest that such mechanisms may be common among pathogens and parasites.

## Prediction 3: Attack against negative regulators imposes diversifying selection, favoring diverse and often rapidly evolving gene families of negative regulators

Hosts gain by altering the specific sequences of their negative regulators to avoid recognition or mimicking by pathogen effectors. Negative immune regulators that are under attack may form diverse gene families and evolve relatively rapidly. Few prior studies have focused explicitly on the diversity and evolutionary rate of negative regulators.

In *Drosophila*, Lee and Ferrandon [33] note that “A striking finding of the cell line screens was the large number of IMD pathway negative regulators that were discovered, as compared with positive regulators.” Across 12 species of *Drosophila*, genes involved in the response signaling cascade tended to be more conserved than recognition proteins, but those that functioned primarily in modulating the immune response showed evidence for positive selection far above the genomic average [34].

The nature of diversifying selection depends on the specificity of mechanisms by which attackers manipulate host immunity. In human immunity, attack against negative immune regulators may be relatively nonspecific, in the sense that the attackers may not be differentially targeting molecules based on sequence variability. By contrast, for the small RNAs that regulate immunity in plants, insects, and other organisms, the RNA sequences and the mechanisms of manipulation are likely to be highly specific. The more sequence-specific the mechanisms that attackers use to manipulate negative immune regulators, the stronger the diversifying selection will be.

## Prediction 4: Strongly opposed positive and negative regulators increase the probability of misregulation and the heritability of immune-related disease

Regulation by strongly opposed triggers and repressors is prone to failure ([35] discusses opposing regulation and disease). The evolutionary tuning of immune control by opposed triggers and repressors is particularly challenging. The intensity of attack and the specific kinds of attack vary widely over time and space. Strong, fluctuating selection likely maintains genetic variability within populations for triggers and repressors.

Plants may use miRNAs to tune the threshold for the triggering of immunity by the NLR pattern recognition receptors. Canto-Pastor and colleagues [36] found that varying miRNA expression quantitatively altered the susceptibility of plants to infection. This potential for quantitative variation in the sensitivity of the triggers likely leads to heritable genetic variation in the speed and intensity of the initial immune response. It would be interesting to learn more about the later-acting negative regulators of immunity that control false alarms set off by the NLR triggers and the potential for quantitative variability in those negative regulators.

Similarly, constitutively expressed inhibitors of the type I IFN response in human immunity may “determine a threshold of activation of an antiviral response” [37]. A quantitively regulated threshold of sensitivity is likely to be associated with heritable quantitative variability in the triggers of immunity.

Genetic variability in triggers and negative regulators can lead to mismatches in the opposing regulatory components of innate immunity and various types of immune-related disease. One possibility concerns the various immune-related disorders associated with misexpression of type I IFN [37]. Another possible example concerns the quick trigger against potentially foreign DNA by cGAS, which is susceptible to autoimmunity [10]. In the cGAS response, misregulation and immune-related disease arise in spite of the many negative regulators of the cGAS cascade.

Our hypothesis and these preliminary examples suggest that further study of genetic variability in the positive and negative regulators of immune cascades may lead to interesting insights about the heritability of immune misregulation.
